# Microcrystallization Effects Induced by Laser Annealing in Cr-Al-C Ion-Beam-Sputtered Films

**DOI:** 10.3390/nano12234136

**Published:** 2022-11-23

**Authors:** Ovidiu Crisan, Alina Daniela Crisan

**Affiliations:** National Institute for Materials Physics, P.O. Box MG-7, 077125 Magurele, Romania

**Keywords:** thin films, ternary compounds, microcrystallization, laser annealing, structural phase transformation

## Abstract

The microcrystallization effects induced by the real-time laser annealing in Cr-Al-C ion-sputtered films with an off-stoichiometric composition are studied. The laser annealing has been performed during Raman experiments with tunable laser power densities. Morphostructural changes induced during laser annealing were investigated by scanning electron microscopy. It has been proven that real-time laser annealing in the high-laser-power-density mode promotes quite clearly the formation of nanograins through surface microcrystallization. Detailed Raman analysis allowed for the observation of the optical modes that unequivocally identifies the low-symmetry 211 MAX phase in both low- and high-power-density modes. Such findings confirming the microcrystallization as well as the stabilization of the grain boundaries by carbon nanoclustering are confirmed by *X*-ray diffraction results, where the single-phase hexagonal 211 was unequivocally proven to form in the high-laser-power-density mode. The microcrystallization via laser annealing was also found to be beneficial for the elastic behavior, as the hardness values between 16 and 26 GPa were found after laser annealing, accompanied by a significantly high Young’s bulk modulus. Such large values, larger than those in bulk compounds, are explicable by the nanometric grain sizes accompanied by the increase of the grain boundary regions.

## 1. Introduction

There has been a surge of interest lately for investigating Cr-Al-C, especially for potential applications as thermal barriers [1,2]. The compound comes from a large class of ternary nitrides and carbides, the MAX systems (M—transition metal, A—metalloid, X—nitrogen or carbon). These systems were proven [3,4] to exhibit both metallic and ceramic features. Their particular nanolaminate structure allows them to show good thermal conductance, high electrical conductivity, being at the same time hard and easily processable, and strongly oxidation-resistant. Fabricated in the shape of films or coatings, the ternary compound has applicability in magnetic bearings or in the autonomous transport domain powered by magnetic levitation. The Cr-Al-C ternary compound’s most stable crystalline phase is the Cr_2_AlC [5,6,7,8,9] hexagonal phase, with the *P63/mmc* space group. The unit cell is made of two Cr_6_C octahedra separated by an aluminum atoms plane. Several methods were employed for the fabrication of the Cr-Al-C films and coatings, either from targets made of individual elements [6,7] or compound targets made of alloys of CrAl with various stoichiometries with carbon additions [8,9,10,11]. The nonequilibrium method of alloying, employing high-vacuum conditions, has also been used for making other nanostructured materials [12,13,14]. Films made of Cr-Al-C were investigated for their potential use as absorbers for Q-switched lasers [15] and for use as shielding components for electromagnetic radiations [16]. In its bulk form, or subjected to sintering procedures, this system was investigated for other industrial domains [17]. Some authors studied the effect of changing the synthesis parameters onto the films’ tribology and oxidation behavior [18,19,20], while others considered the influence of ion beam annealing onto films of Cr-Al-C [21].

We had previously studied the carbon nanoclustering effects in Cr-Al-C systems [22,23,24] and we have proven that a single 211 MAX phase can be achievable at temperatures as low as 450 °C. At the same time, we demonstrated that the elastic behavior of Cr-Al-C [24] is dependent on the crystallization procedures. In that sense, it was shown that in-air annealing produces a chromium oxide layer at the films’ surfaces, thus preventing further corrosion.

Several authors, when investigating Cr-Al-C and MAX films in general, have pointed out [8,9,25] that there is a need of quite high temperature for heating the substrates to obtain single MAX phases, usually above 650 °C. Such high temperatures introduce an extra processing sequence which may impede the use of such materials in industrial applications. Not only that, but it also restricts the available choices for materials to make substrates to only those proven to be stable above 650 °C. Obtaining crystallization by the subsequent annealing of the films occurs most of the time with an associated, inherent oxidization process. The present paper demonstrates a way to circumvent this problem of achieving crystallization with too-high costs for industrially relevant processes and with the inherent side-effect of oxidation during annealing.

In this paper, we show that real-time microcrystallization can be achieved during the laser annealing procedure that is done during the films’ characterization via Raman spectroscopy. The laser annealing procedure has been widely used for recrystallization and surface treatments in many applications. For instance, Son et al. [26] investigated the laser melt annealing of amorphous silicon (a-Si) and the subsequent recrystallization of a-Si for successfully implementing vertical flash memory devices. In another work, Novotny et al. [27] used pulsed laser annealing on Eu-doped ZnO, TiO_2_, and Lu_2_O_3_ thin films, and they measured in situ the photoluminescence and transmittance of the devices during the annealing. Latronico et al. [28] investigated the skutterudite thin films and showed that lowering the thermal conductivity can be achieved by laser annealing via adding scattering centers as well as increasing the number of defects. The laser treatment of materials was also successfully used in [29], where annealing by laser-heated electrospinning was proven to be efficient in producing ultrafine fibers. Heat transfer was even proven to be successful in changing the magnetic state of thin films in [30], where it was shown that cutting diamond crystals with a laser (532 nm wavelength, 0.5 mJ energy, 200 ns pulse duration at 15 kHz) produced a 20 nm thick surface layer with a magnetic order at room temperature. Laser-induced temperature changes might be suitable for the in situ monitoring of the repair of defective sites in fused-silica optical components [31]. Structural phase transformation induced by the laser irradiation of surfaces was also reported in [32], where it was pointed out that, whereas the conventional thermal heating of TiO_2_ samples resulted in the formation of only the anatase phase, laser annealing exclusively produced on the same as-deposited sample the rutile phase.

The laser annealing effects on the films’ microstructure are investigated in two different annealing regimes: the low-laser-power-density (LLPD) and the high-laser-power-density (HLPD) modes. The main structural properties of the Cr-Al-C films are investigated, and the microcrystallization occurring during the HLPD irradiation regime is shown to promote the formation of the single 211 MAX phase, this being accompanied by an increase of the elastic properties and the hardness features of the Cr-Al-C films.

## 2. Materials and Methods

### 2.1. Synthesis

**Figure 1:**
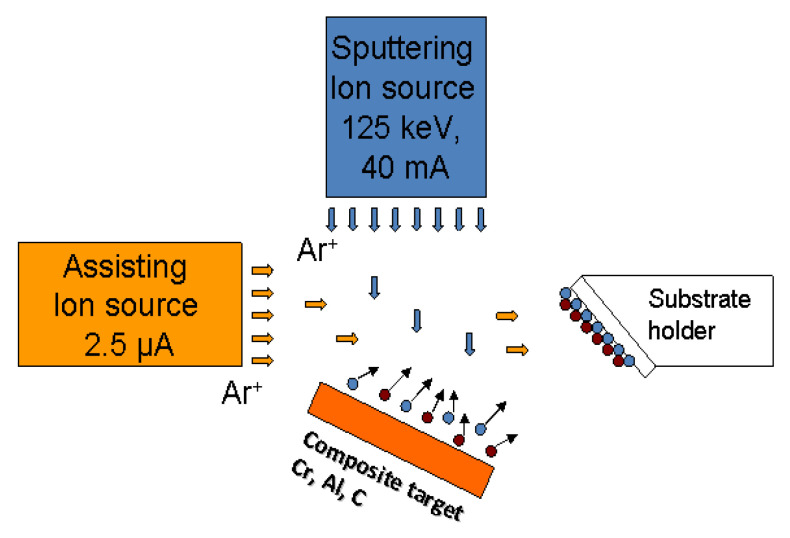
Schematics of the ion-assisted deposition process. Top-mounted ion source sputters the target while laterally mounted assisting ion source furnishes additional energy that help atoms to better bond on the substrate.

Several samples of the Cr-Al-C films have been synthesized following a cosputter deposition method using a complex dual-ion beam facility. The synthesis has been performed in a high-vacuum (HV) chamber, having an initial pressure of 5 × 10^−6^ mbar. The dual facility has two ion beam sources mounted on perpendicular directions, one to another: the sputtering ion source, mounted on top of the facility, oriented vertically, and the assistive ion source, mounted on the side of the facility, oriented horizontally. Both the target and the substrate holder are mounted obliquely from the ion beam directions. The schematic of the deposition process is given in Figure 1. The target is mounted on the bottom of the facility, having its plane oriented under an angle of about 57° with respect to the vertical, facing the substrate holder, which is mounted on the other side of the facility, almost parallel to the target plane. All sample films are deposited using both sputtering and assisting ion sources. The target is a compound one, made of a pure Cr (99.99%, from Alfa Aesar GmbH, Karlshruhe, Germany) plate which is partially covered by graphite (from Sigma-Aldrich, Burlington, MA, USA) as well as Al (99.9% from Sigma-Aldrich Chemie GmbH, Taufkirchen, Germany). The specific areas covered by each of the elements accurately provides the needed stoichiometry of the whole film. The sputtered areas of the elemental targets are fully adjustable, allowing us to finely tune the desired elemental percentages. During the synthesis sequence, the compound target is sputtered from the sputtering ion source—top-mounted—by 1.2 keV Ar^+^ ions, with the 75 mm ion beam having a current of 125 mA and an acceleration voltage of 1.5 kV. The assisting ion source, laterally mounted, as in the scheme from Figure 1, has a much smaller current of only 2.5 μA, and runs simultaneously with the sputtering source and has essentially 3 main roles: (i) it helps remove the gas contaminants from the substrate during deposition, (ii) it transfers extra energy to the depositing atoms, which gives them more surface mobility, allowing them to move to different nucleation sites and better bonding, (iii) and it gives a local added energy to the atoms to move, as if they are hotter than they normally are, taking into account the cooling rates of about 10^14^ K s^−1^. It conclusively helps produce denser and more compact films, as well as compounds and phases which would not normally be possible at the ambient temperature.

Thin films of Cr-Al-C with various stoichiometries have thus been deposited onto Si (100) and high-speed steel substrates. The deposition time and rate was kept almost constant so as to ensure a similar thickness for all samples of about 150 nm. The pressure parameters for a typical deposition sequence records an Ar pressure of 3.5 × 10^−4^ mbar in the sputtering chamber. Before deposition, the target is cleaned by ion sputtering with Ar^+^ ions, a procedure which takes 30–45 min. The assistive ion source can operate using a current between 2.5 and 30 μA; however, during the synthesis sequences, the ion assistance with the lowest current available was chosen (2.5 μA). This is because an enhanced assisting ionic flux, with higher currents, would normally require constant readjustments of the target configuration to ensure that the desired stoichiometry is preserved.

### 2.2. Characterization

The complete structural characterization of the samples has been performed using a variety of experimental tools. The elemental composition of the sputtered samples has been established using energy-dispersive *X*-ray spectroscopy (EDS-EDX). The structure and morphology of the dual-sputtered samples, as-synthesized and laser annealed, have been investigated using *X*-ray diffraction (XRD), scanning electron microscopy (SEM), and Raman spectroscopy. The SEM images were taken using an EVO 50 XVP microscope (Carl Zeiss GmbH, Oberkochen, Germany). XRD analysis was done with a D8 Advance powder diffractometer from Bruker AXS GmbH, Karlsruhe, Germany. Measurements were taken using Cu Kα radiation (λ = 0.154 nm). The geometry used for acquiring the XRD was the classical θ–2θ with an incidence angle of 1.5° in an angular interval from 20° to 90°. From the XRD data, by using full profile analysis, lattice parameters and average grain sizes were obtained. The software used for the full profile analysis was MAUD (materials analysis using diffraction). Raman spectroscopy was realized using a Renishaw InVia facility from Renishaw plc, Gloucestershire, UK. This spectrometer was used with data collection in the backscattering configuration. The experimental procedure employed an Ar laser of 512 nm wavelength of various laser powers. The duration of the irradiation was kept at 30 min and the sample was not preheated before the laser annealing. For recording the Raman spectra, we used the rotating surface scanning technique with a schematic setup similar to that reported in [33]. The beam was focused on the sample surface using a 50× objective. The 50× objective produced a large spot size of about 830 nm in diameter. Such a large spot size on the sample surface allowed us to perform our study where the effects of different laser power densities on the structure of irradiated samples were studied in detail in the present paper. For this purpose, the laser power densities were adjusted between 2.5 and 6 mW/cm^2^. In order to separate the microcrystallization effects induced by the various laser power densities, we convened to separate the laser power densities into two intervals. Between 2.5 and 4 mW/cm^2^, the irradiation regime is called the low-laser-power density (LLPD) and between 4.5 and 6 mW/cm^2^, the irradiation regime is called high-laser-power density (HLPD). The irradiation under the HLPD regime has been done so as to ensure that the mechanism of annealing is thermal and so as to ensure the onset of the crystallization processes. Moreover, the absorption coefficient at the 512 nm laser wavelength was estimated for the CrAlC max films to be between 9.5 and 12.7 cm^−1^, depending on the stoichiometry of the films, while the thermal conductivity ranged between 5.5 and 6.2 W m^−1^ K^−1^. We estimated that the irradiance was not high enough to melt the film; however, the expected temperature after annealing was between 600 °C and 620 °C, which was sufficient to induce the crystallization of the film. The spectral resolution of the obtained line-widths and peak positions was within 2 cm^−1^. All the obtained Raman spectra have been fitted using a line profile corresponding to a Gaussian–Lorentzian distribution with a special software, suitable for the accurate deconvolution of Raman spectral peaks (PeakFit 4.12, Systat Software GmbH, Erkrath, Germany). This kind of fitting procedure allows for the proper deconvolution in the case of highly overlapping peaks, as is the case in such multiphase materials.

## 3. Results

### 3.1. Composition and Morphology

In order to obtain samples with various stoichiometries, the irradiated area covered by each target element was carefully measured and tailored. Therefore, through this method, we are able to obtain several compositions, with the Cr to Al to C ratio varied accordingly. The adjustable irradiated target area of each element resulted in various relative amounts in the ternary alloy. These relative amounts have been verified using the EDX module of the SEM. For the purpose of the present study, a film with the composition Cr_56_Al_21_C_23_ has been cosputtered. The real chemical composition was determined using EDX and was found to be close to the nominal composition, within an error of 1.5 at%. The estimated stoichiometry errors, averaged over all three determinations, were about 0.12 at%.

In order to investigate the effect of laser annealing on the microstructure of the as-obtained thin films, we performed Raman spectroscopy measurements in both the LLPD and HLPD regimes. Besides recording the resulting Raman spectra for each of the investigated sample, we also comparatively investigated the structure of the films before and after the laser annealing. This was done for a better and clearer insight on the microcrystallization effects induced via laser annealing during the real-time Raman measurement. The duration of the recording the Raman spectra has been set for convenience to be 30 min, the same for all the investigated samples

**Figure 2:**
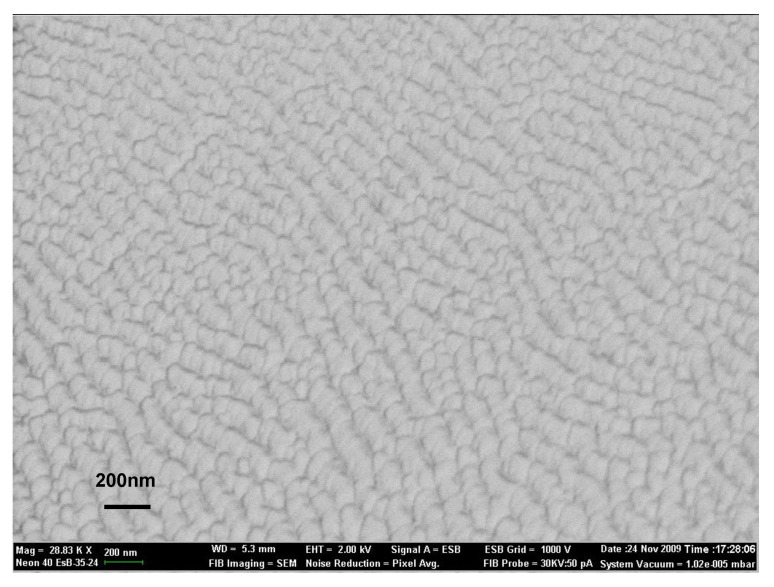
Area of the surface of Cr_56_Al_21_C_23_ as-deposited thin film. Rastering effects due to the assisting ion source are revealed by SEM images.

**Figure 3:**
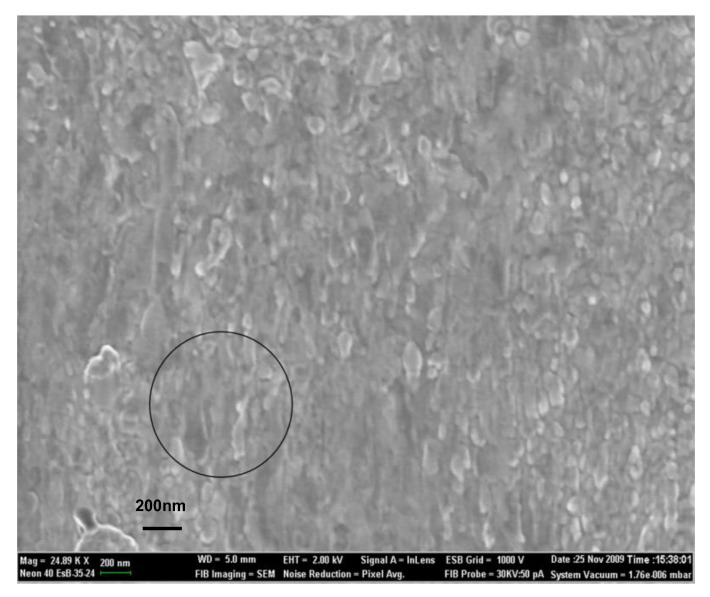
Area of the surface of Cr_56_Al_21_C_23_ after laser annealing in HLPD regime. Occurrence of small crystallites is observed. Approximate position of the laser spot is shown.

For the morphology investigations of the thin films, the SEM images have been recorded on the surface of both the as-deposited and laser-annealed samples. Figure 2 and Figure 3 present the SEM images of the sample Cr_56_Al_21_C_23_ as-deposited and laser annealed in the HLPD regime, respectively.

It can be observed that the as-deposited film presents a rather uniform and homogeneous surface, with waving effects quite regularly dispersed, as observed on the whole investigated area. It is considered that these regular surface waves are in fact due to the rastering effects induced by the assisting ion source during deposition of the film. Different features are observed for the laser-annealed area. The circle in the Figure 3 represents the approximate position and size of the laser beam. Here, a more inhomogeneous surface with the occurrence of spots of different contrast, some of them quite regularly round-shaped, can be observed. These spots witness the occurrence of nanograins of a quite small scale. By using a histographic method, we have estimated the average grain size in the SEM image to be about 59 ± 5 nm. Besides these small crystallites, some larger cluster aggregates are also observed, again due to the laser-annealing effects. The nanograins observed in the laser-annealed sample present similar contrast effects, with no other different texturing observed. This shows a quite homogeneous annealing procedure, giving rise to similar nanograins, most probably with the same crystal structure.

### 3.2. Raman Analysis

The thin films have been subjected to Raman analysis under the two laser-irradiation regimes: LLPD and HLPD. The Raman scattering spectra have been recorded at 120–2000 cm^−1^ for a fixed duration of 30 min. The whole investigated wave numbers interval has been split into three typical regions, which is a separation that is customarily made in the interpretation of the Raman spectra. It is known that the Cr-Al-C phase structure can be assigned to the D^4^_6h_ space group. It has been reported [34,35] that the Cr-Al-C system presents four optical modes. Three of these modes are Raman-active, while the last mode is both infrared-active and Raman-active [36].

As we have shown previously [23], and as also mentioned before, the Raman spectra of Cr-Al-C are separated into three Raman regions: the low (0–500 cm^−1^), the intermediate (500–1000 cm^−1^), and the high (1000–1800 cm^−1^) regions. It is worthwhile noting that, in the previous literature, not more than one or two of these regions were customarily investigated. In [34], the Raman investigation was conducted only until 800 cm^−1^, while in [37], only the Raman modes between 1200 and 1500 cm^−1^ were shown for Cr_2_AlC; moreover, these peaks belonging to the third region were assigned not to the MAX phase itself but to the carbon bands. It is now usually accepted that the low-spectral region encompasses Raman peaks that are attributable to the M_2_AX phases. Since 211 structures have as parent the D^4^_6 h_ space group, the three optic modes, Raman-active, are due to *A_g_* + 2*E_2g_* levels, and the fourth, infrared and Raman-active, is due to *E_g_* level [34].

**Figure 4:**
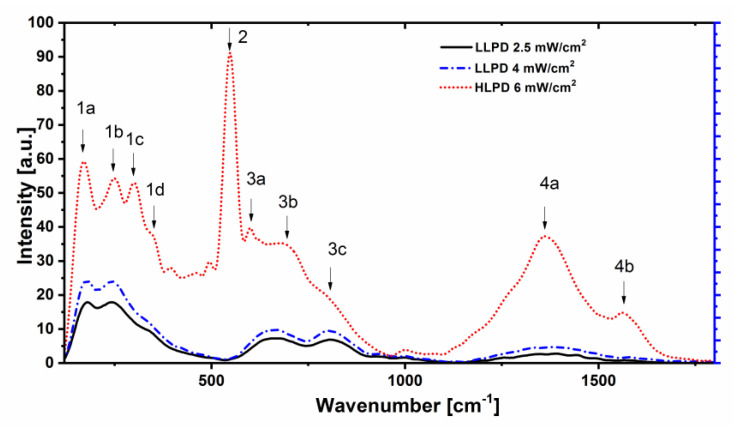
Raman spectra of the sample 1 recorded in three different laser-annealing regimes: LLPD at 2.5 mW/cm^2^, LLPD at 4 mW/cm^2^, and HLPD at 6 mW/cm^2^.

In the Figure 4, we represent the Raman spectra of sample 1, recorded in three different laser annealing regimes: LLPD at 2.5 mW/cm^2^, LLPD at 4 mW/cm^2^, and HLPD at 6 mW/cm^2^. It can be seen that the low-spectral region has some large, convoluted Raman peaks in the case of the LLPD-recorded spectra, very large but with smaller intensity Raman peaks in the intermediate spectral region, and almost no Raman peaks in the high-spectral region for both the investigated LLPD spectra: at 2.5 and 4 mW/cm^2^.

The situation is drastically changed in the case of the HLPD Raman experiment at 6 mW/cm^2^. In this case, much more intense and sharp Raman peaks are observed. The sharp peaks observed in the low-spectral region have larger intensities than in the case of the LLPD mode and they are sharper. The usual notation for these peaks appearing in the low-spectral region is *1a*–*1d* peaks, and they constitute the signature of the occurrence of the crystalline MAX phases. The Raman peaks attribution from the low-spectral region is consistent with other reports from the literature [23,34,35,36,37].

In the intermediate spectral region for wavenumbers between 500 and 1000 cm^−1^, the MAX phase materials customarily show two broader peaks of lower intensity. In our notation, these peaks are denoted as *3a–3b*. Such Raman modes in the intermediate region are also observable in the MAX systems, and they are usually not attributed to any other binary phases in the ternary compound. In several other papers on Cr-Al-C systems, these peaks have been possibly assigned to other Raman-active modes in the Cr_2_AlC phase [34,35]. Apart from these modes, a very intense, sharp peak appears at about 548 cm^−1^. In agreement with previous reports [21], this quite intense and sharp peak has been assigned to the occurrence of chromium oxides, notably Cr_2_O_3_. All the values recorded for the Raman peaks in the spectra are listed in Table 1.

The HLPD Raman spectrum presents in the high-spectral region, listed between 1000 and 1800 cm^−1^, two very large and intense Raman lines situated around 1362 and 1568 cm^−1^, respectively. In agreement with the existing literature [23,38,39,40,41], these large and intense peaks from the high-spectral region are customarily attributed to the D- and G-bands of carbon, respectively. Such Raman peaks are occurring in the MAX compounds where there is an imbalance in the stoichiometry towards carbon excess.

### 3.3. Structural Analysis

Most of the physical properties that made the MAX phases interesting for applications are obtained in the crystalline state of the deposited films. Since films are sometimes obtained in an amorphous-like or solid-solution state, and higher-substrate temperatures or postdeposition annealing is frequently required to obtain a crystalline structure, it is important to characterize structurally the deposited films to check the crystal structure as well as phase composition. The structural analysis allows verifying the amorphous or crystalline state of the as-deposited films. One of the signs for crystallinity is represented by the broadness of the observed Raman peaks. To certify the formation of the Cr_2_AlC MAX phase during annealing in [18], Raman analysis was employed. The big bump in the Raman spectrum was found to be a proof of the initial amorphous state of the sample, while after annealing, *1a*, followed by *1b* convoluted with *1c* and, respectively, *1d* Raman peaks were observed in [18] and considered evidence of the crystallization of the sample and occurrence of the 211 hexagonal phase.

**Figure 5:**
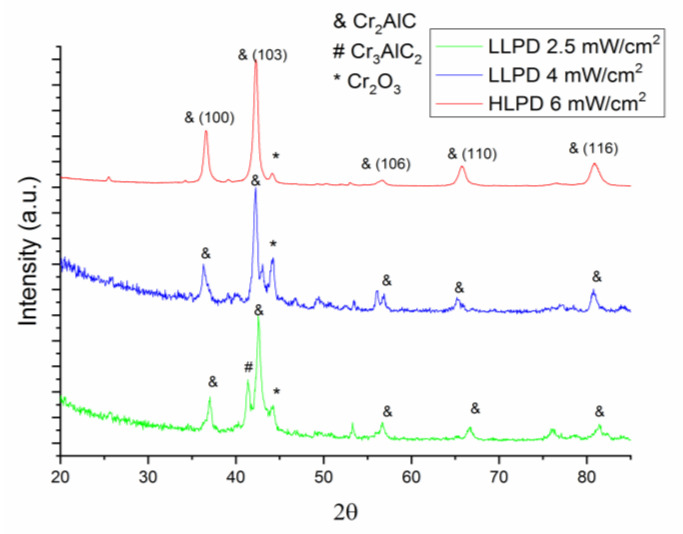
XRD patterns of the films as-deposited (**bottom**) and films annealed at 700 °C (**top**).

Notwithstanding that, more direct evidence about the crystal state of the sample is given by the XRD analysis. For this analysis, we have measured using *X*-ray diffraction the samples that have been subjected to the LLPD and HLPD laser annealing during the Raman experiments in order to investigate exactly the degree of crystallization induced by the laser annealing itself. XRD measurements have thus been performed onto the samples after the LLPD and HLPD laser annealing. These obtained structural data have been analyzed using a full-profile fitting procedure. In that scope, the obtained XRD data have been fitted using materials analysis using diffraction (MAUD) software [42], which follows a method of refinement similar to Rietveld-type analysis, which has been improved and adjusted for use with topologically disordered alloys through the inclusion of a pair distribution function [43]. The method to separate the overlapping broad Bragg peaks, as happens in polycrystals and topologically disordered materials [44], follows a fitting algorithm that has been used and described in other previous papers [45,46,47,48,49,50,51,52,53]. We have shown, in Figure 5, the *X*-ray diffractograms for the samples subjected to the three different laser-annealing procedures: LLPD at 2.5 mW/cm^2^, LLPD at 4 mW/cm^2^, and HLPD at 6 mW/cm^2^. The XRD of the LLPD at 2.5 mW/cm^2^ sample presents the formation of a mostly crystalline pattern, as proven by the quite well-formed, sharp, and intense Bragg peaks. Here, most of the identified Bragg peaks have been assigned to the hexagonal Cr_2_AlC phase. Besides the Bragg lines assigned to this main 211 phase, in the main Bragg peak of the Cr_3_AlC_2_ phase, a higher symmetry 312 MAX phase is visible at around 42° in 2θ, but with lower intensity. The pattern recorded for the sample laser-annealed LLPD at 4 mW/cm^2^ shows a rather similar allure, as the same Bragg lines are identified as in the case of the sample laser-annealed at 2.5 mW/cm^2^.

The XRD pattern for the HLPD sample at 6 mWcm^2^ is, however, different. Here, the Bragg lines are better defined and narrower; moreover, all the other satellite peaks not belonging to the main 211 MAX phase have disappeared. This exception makes the small Bragg peak attributable to the hexagonal Cr_2_O_3_ phase. The existence of the chromium oxide Bragg peak in the XRD pattern confirms well the results obtained in the Raman analysis, where we have also unambiguously identified the presence of a chromium-oxide-related Raman peak, labeled as peak 2 in the Table 1.

The full-profile fitting with MAUD has allowed the identification of the most important lattice parameters of the Cr_2_AlC phase in all three samples. The fitting has taken into account not only the position of the Bragg peaks and its full width at half-maximum, but also the asymmetry of the Bragg profile, marked through the mixing parameter, characteristic for the pseudo-Voigt function used for the Bragg lines fitting. The results of this fitting, lattice parameters, hexagonal unit cell volume, the average crystallite size, as well as the microstrain, parameters that have been obtained by using MAUD, are listed in Table 2.

It can be observed that the lattice parameter *a* decreases with increasing the laser power density, with about 1.2% more for the HLPD sample than the LLPD sample. At the same time, the lattice parameter *c* diminishes more sharply (5.4%) in the HLPD. There is a phenomenon quite often encountered in the microcrystallization of ternary compounds which are bound to exhibit a sequence of steps, as follows: firstly, the primary crystallization, characterized by the creation of nucleation sites, whereby nucleation sites cease to be created and instead start to grow into the so-called secondary crystallization, which is basically a nucleation-to-growth model with differently manifesting activation rates for nucleation and for growth. In this case of microcrystallization induced by the real-time laser annealing, as the crystallization passes into the secondary stage, the ordering of the crystal is prone to occur along the *c*-axis. This ordering is followed by the formation of the main building blocks made of Cr_6_C octahedra, layered alternatively with Al atomic layers, to form the nanolaminate structure of the Cr-Al-C films [5]. Following the overall decrease of the lattice parameters, the calculated unit cell volume also diminishes for the HLPD laser-annealed sample. From the results in Table 2, it can be observed that the average crystallite size constantly increases upon increasing the laser power density. From the value of 34 nm calculated for the LLPD for the 2.5 mW/cm^2^ sample, the average crystallite diameter increased up until 56 nm for the HLPD sample. It is worthwhile to note that the value calculated after the MAUD fitting of the XRD patterns is in very good agreement with the mean grain diameter value of 59 nm, measured via the histographic method in the SEM image of Figure 3. Such good agreement proves the accuracy of our methods for the estimation of the average crystallite size by two different procedures. The effectiveness of the microcrystallization obtained through laser annealing is also proven by the decrease of the lattice microstrain observed for the case of the HLPD sample compared with both of the LLPD samples. In the HLPD sample at 6 mW/cm^2^, the lattice microstrain is lowered by almost 61% compared to the lattice microstrain observed in both samples laser annealed in the LLPD mode. This ensures that laser annealing is also an effective method to reduce the microstrain observed at the crystal lattice level in the not-completely ordered 211 structures in the intermediate stages of annealing, where additional elements can bring defects in the 211 hexagonal lattice, defects that are quantified in a larger value and globally measured for the lattice strain.

### 3.4. Elastic Properties

**Figure 6:**
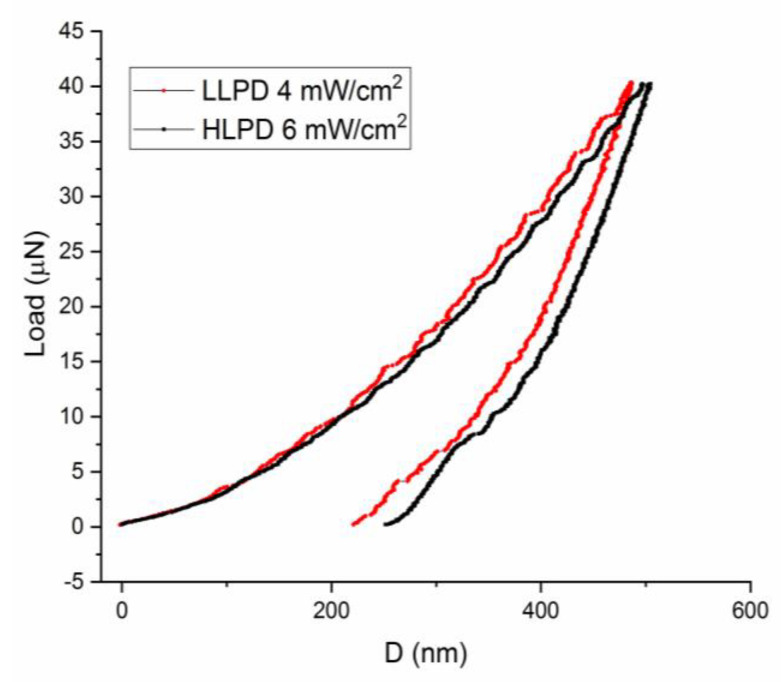
Load-displacement curves recorded for laser-annealed LLPD samples at 4 mWcm^2^ as well as for the sample laser annealed in the HLPD mode at 6 mW/cm^2^.

The effectiveness of laser annealing in improving the properties of the Cr-Al-C films can also be proven by investigating the elastic properties of the film. To study these properties, nanoindentation experiments using a G200 nanoindenter device (Keysight Technologies France SAS, Les Ulis, France) with a Berkovich nanoindenter tipm have been performed. In the experiments performed at room temperature, the load–displacement graphs have been recorded on samples laser annealed in the LLPD mode at 4 mWcm^2^, as well as on the sample laser annealed in the HLPD mode at 6 mW/cm^2^. The corresponding load–displacement curves for the 40 μN loading weight are shown in Figure 6, while the corresponding results obtained from the analysis of these curves, i.e., the hardness *H* and the reduced elastic modulus *Er*, are drawn in Table 3.

It can be seen that there is a quite large increase of the hardness in the laser-annealed HLPD sample at 6 mW/cm^2^ compared with the LLPD mode samples. The calculated hardness values for the laser-annealed samples are between 16.72 and 26.25 GPa, while the elastic modulus, an average value calculated via a model that takes into account the reduced bulk modulus with an empirically found proportionality constant, as described in [54], ranges between 217 and 261 GPa. In the Table 3, we depict the calculated values for the hardness and the reduced bulk modulus for the laser-annealed LLPD samples at 2.5 and 4 mW/cm^2^, as well as for the sample laser annealed in the HLPD mode at 6 mW/cm^2^.

Our calculated results are in agreement with the results from the existing literature. For instance, the same elastic parameters, the hardness and the elastic modulus, are reported in [54] to be 13.5 GPa and 268 Gpa for Cr_2_AlC thin films. It is to be noted that the hardness values registered for the thin Cr-Al-C films are, most of the time, significantly larger than their counterparts recorded in the bulk MAX phases. In the case of the Cr_2_AlC bulk material, this value does not generally exceed 3.5 GPa [55,56]. This large inconsistency is explained in [54] as being due to the average grain size diminishing towards tens of nm, accompanied by a sharp increase of the grain-boundary density compared to the bulk MAX materials, where average grains are usually in the µm range.

## 4. Discussion

To explain the occurrence of the D- and G-bands in the Raman spectra of the HLPD-annealed CrAlC, a phenomenological model proposed in [38] described the excess of carbon in the 211 thin films as having a disordered structure made of covalent sp^2^- and sp^3^-bonded carbon. In this respect, the model assumes that the Raman peak assigned to the G-band occurs because of the shortening of the C-C bond in the pairs of sp^2^ atoms, while the Raman peak assigned to the D-band may be attributed to the *A_1g_* breathing modes. Such values were reported in [38] as occurring at 1360 and 1560 cm^−1^, respectively, and in [39], the same broad Raman peaks were found to occur at about 1380 cm^−1^ and 1560 cm^−1^, respectively. In our determination, in the HLPD mode, these peaks appear at 1362 and 1568 cm^−1^, respectively; therefore, we can conclude that we find good agreement with the previously reported data. There were some alternative potential causes [9,20,41] for the occurrence of the dominant D- and G-bands in the Raman spectra of the 211 films. In [41], it was suggested that carbon clusters of quite large dimensions might have preferential orientation, and in [20], it was inferred that the whole high-spectral region encountered in the Raman spectra can be assigned to diamond-like carbon clusters having large grain boundaries made of sp^2^-bonded carbon. The grain size in the carbon-containing films can also be extracted from the Knight and White empirical expression [57] that relates the average grain size to the ratio between the D- and G-band intensities, or lately from the generalized formula [58]:

$$L_{a}\left(nm\right)=\left(2.4\times 10^{-10}\right)\lambda_{l}^{4}{\left(\frac{I_{D}}{I_{G}}\right)}^{-1}$$

where *L_a_* is the average grain size, λ_l_ is the laser wavelength, and *I_D_* and *I_G_* are the integrated intensities of the D- and G-bands, respectively. Taking into account the integral intensities of the Raman peaks associated to the D- and G-bands, calculated from the Raman spectrum of the HLPD annealing case, we have derived the average grain size *L_a_* to be around 51 ± 7 nm. The very-good agreement with both the SEM observations and the results of the fitting *X*-ray diffractograms may be noted. This proves the accuracy of our determinations as well as the crystallization effects of the laser annealing, not only on the MAX phase grains, but also on the graphitization of the intergranular carbon-rich regions.

## 5. Conclusions

The present paper proposes a study of the microcrystallization effects induced by the real-time laser annealing of Cr-Al-C films with the off-stoichiometric composition 56:21:23. The films have been cosputtered from a dual ion source in ultra-high-vacuum conditions, and the laser annealing has been performed during the Raman experiment under variable laser power densities. The effects of laser annealing were quantified taking into account two different modes: the low-laser-power-density (LLPD) mode, for power densities between 2.5 and 4 mW/cm^2^, and the high-power-laser-density (HLPD) mode, for power densities between 4.5 and 6 mW/cm^2^. The morphostructural changes in the ternary thin films of the Cr_56_Al_21_C_23_ composition have been investigated by scanning electron microscopy, where it was seen that the HLPD mode of annealing promotes microcrystallization quite clearly, as observed via the many nanograins of about 59 nm average size which appear after the HLPD annealing. Detailed Raman analysis, which was accompanied by a fitting–deconvoluting procedure, allowed for the observation of three different Raman spectral intervals. Two of these Raman spectral intervals contain the optical modes that unequivocally identify the low-symmetry 211 MAX phase in both the LLPD and HLPD modes, with the last Raman spectral interval indicating the effects of the carbon nanoclustering forming grain boundaries between the 211 phase nanograins solely in the HLPD mode of annealing. The results, confirming the microcrystallization and stabilization of the grain boundaries by carbon nanoclustering, were further supported by the *X*-ray diffraction results, where the evolutionary state of the microstructure was monitored and the hexagonal 211 phase was unequivocally proven to form in the HLPD annealing mode as a single phase in the film. Both lattice parameters and unit cell volume are shown to decrease upon the HLPD annealing as a consequence of the nucleation, followed by grain growth during microcrystallization. The microcrystallization via laser annealing is also found to be beneficial for the elastic properties of the films. Large hardness values between approximately 16 and 26 GPa are deducted from the nanoindentation determinations. These significantly large values, larger than what is found in bulk compounds, can be justified by the nanometric size of the grains and the increase of the volume occupied by the grain boundaries.

## Figures and Tables

**Table 1:** Values recorded for the Raman peaks observed in the three Raman experiments in LLPD and HLPD annealing modes.

Peak no.	*1a*	*1b*	*1c*	*1d*	*2*	*3a*	*3b*	*3c*	*3a*	*3b*
LLPD 2.5 mW/cm^2^	179	242	-	336	-	-	662	810	-	-
LLPD 4 mW/cm^2^	177	241	-	338	-	-	663	809	-	-
HLPD 6 mW/cm^2^	165	248	304	347	548	598	686	805	1362	1568

**Table 2:** The lattice parameters, average crystal size, volume of the unit cell, and microstrain of the 211 MAX phase calculated from the results of MAUD fitting.

Sample	Lattice Parameters			Cryst. Size	Microstrain
	a (Å)	c (Å)	V (nm^3^)	(nm)	(%)
LLPD at 2.5 mW/cm^2^	2.884 ± 0.011	13.42 ± 0.03	0.097	34 ± 4	0.79 ± 0.03
LLPD at 4 mW/cm^2^	2.861 ± 0.008	13.15 ± 0.03	0.093	39 ± 6	0.42 ± 0.05
HLPD at 6 mW/cm^2^	2.848 ± 0.005	12.69 ± 0.04	0.089	56 ± 6	0.31 ± 0.05

**Table 3:** The calculated values for hardness and reduced bulk modulus for the laser-annealed LLPD samples at 2.5 and 4 mW/cm^2^, as well as for the sample laser-annealed in HLPD mode at 6 mW/cm^2^.

Sample	Elastic Properties	
	Hardness H (GPa)	Reduced Modulus (GPa)
LLPD 2.5 mW/cm^2^	16.72 ± 0.76	217 ± 2
LLPD 4 mW/cm^2^	19.37 ± 1.18	225 ± 7
HLPD 6 mW/cm^2^	26.25 ± 0.71	261 ± 9

## Data Availability

The data are not publicly available due to IPR protection measures. Access to part of the data can be granted on a case by case basis, upon request.
